# Partial cricotracheal resection for treatment of subglottic stenosis: complications and outcomes

**DOI:** 10.3389/fsurg.2025.1559943

**Published:** 2025-02-13

**Authors:** Jeroen Meulemans, Laila Mouqni, Noah Ostyn, Davide Di Santo, Greet Hens, Vincent Vander Poorten, Christophe Dooms, Nico De Crem, Paul De Leyn, Ann Goeleven, Pierre Delaere

**Affiliations:** ^1^ Otorhinolaryngology, Head and Neck Surgery, University Hospitals Leuven, Leuven, Belgium; ^2^ Department of Oncology, Section Head and Neck Oncology, KU Leuven, Leuven, Belgium; ^3^ Pulmonology, University Hospitals Leuven, Leuven, Belgium; ^4^ Thoracic Surgery, University Hospitals Leuven, Leuven, Belgium

**Keywords:** cricotracheal resection, partial cricotracheal resection, subglottic stenosis, tracheal stenosis, voice outcomes

## Abstract

**Purpose:**

Subglottic stenosis (SGS) is defined as an obstruction of the subglottic area, potentially extending towards the first tracheal rings. Although endoscopic procedures are frequently preferred as first-line treatment, (partial) cricotracheal resection (PCTR) offers the most durable results. This study aims at reporting and analysing complications and respiratory and vocal outcomes after PCTR.

**Methods:**

For this retrospective cohort analysis, the files of 37 patients with SGS who underwent PCTR in a tertiary referral center were reviewed. Patient- and stenosis-characteristics along with postoperative outcomes and complications were analyzed using descriptive statistics.

**Results:**

The majority of patients were female (95%), which reflects the high incidence of idiopathic SGS in our patient group (89.2% vs. 2.7% postintubation SGS and 8.1% SGS related to systemic inflammatory disease). Most patients presented with a Cotton grade II (35.1%) and III (54.1%) stenosis, with a mean craniocaudal stenosis length of 17.5 mm. The vast majority of patients (89.2%) had undergone previous endoscopic procedures. The most common complication after PCTR was fibrin deposit/granulation tissue formation at the anastomotic site (*n* = 15, 40.5%). Other complications were rare, with anastomotic dehiscence, postoperative haemorrhage and vocal cord paralysis each in 1 patient (2.7%), temporary tracheostomy in 2 patients (5.4%), and postoperative wound infection in 3 patients (8.1%). During follow-up, only 2 patients (5.4%) developed restenosis which was successfully salvaged by endoscopic procedures. No patients were long-term tracheostomy dependent. Post-operative mean peak expiratory flow (PEF) percentage showed a 43.7% increase compared to pre-operative. For the mean increase in maximum inspiratory flow (MIF) at 50% this was 1.3 L/s. VHI (voice handicap index) scores increased significantly from baseline preoperative score of 27.5 (±23.7) to a mean value of 54.9 (±18.7) (p = 0.002) 1-month postoperatively but decreased below preoperative scores after 2 years (22.2 ± 18.1, *p* = 0.036).

**Conclusion:**

PCTR is an efficient treatment for SGS, with low complication rates, a low rate of long-term restenosis and good vocal outcomes.

## Introduction

**Figure 1:**
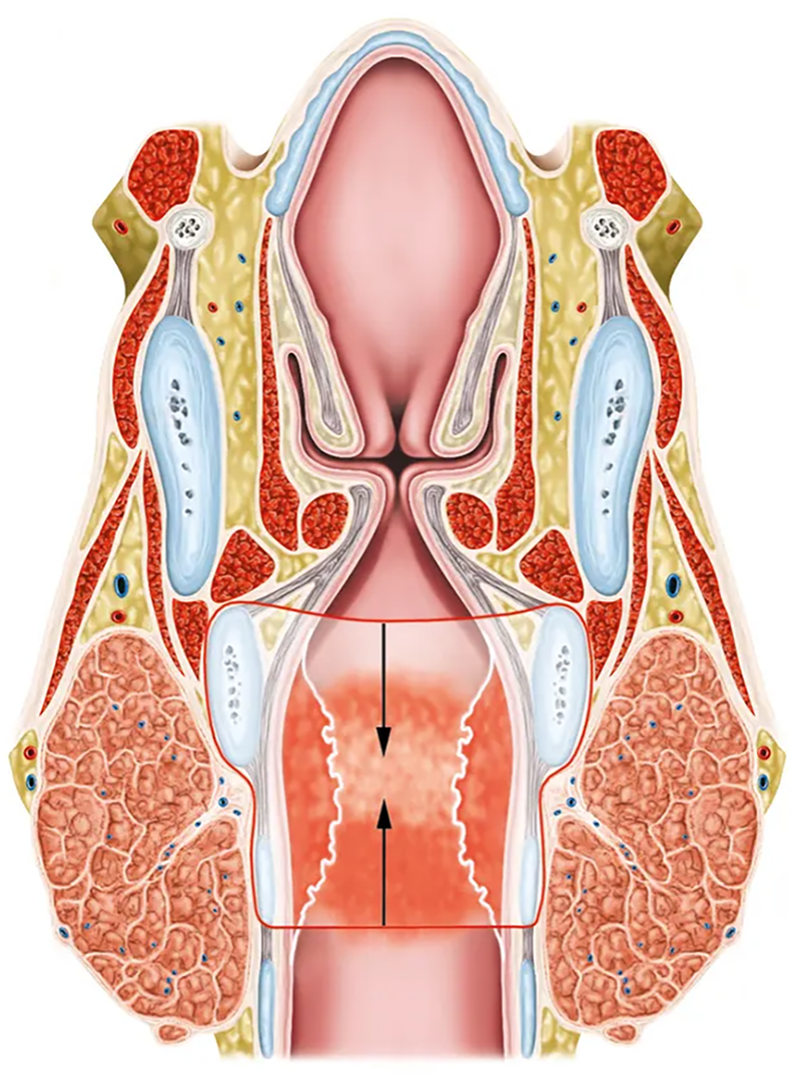
Coronal section of a subglottic stenosis at the level of the cricoid cartilage and the first tracheal ring. The extent of resection during partial cricotracheal resection is indicated in red lines. The arrows show the direction of the anastomosis between the trachea and the posterior cricoid and thyroid cartilages.

Subglottic stenosis (SGS) is a condition that is characterized by a (progressive) obstruction of the subglottic area and potentially the first 2 tracheal rings, causing dyspnoea, stridor, coughing, exercise intolerance and voice complaints (Figure 1) (1, 2). In adults, SGS has multiple etiologies with the most common cause being iatrogenic trauma due to endotracheal intubation. Less frequently, SGS is the result of external or heat trauma, external beam radiotherapy, previous head and neck surgery, upper respiratory tract infections, gastroesophageal reflux disease (GERD) or systemic diseases, such as granulomatosis with polyangiitis (GPA), amyloidosis and Immunoglobulin G4-Related Disease (IgG4-RD) (3–6). A rare but increasingly common variant is the idiopathic SGS (iSGS), defined as a subglottic stenosis of which the underlying cause cannot be determined. iSGS is a slowly progressive, nonspecific inflammatory process that is mainly diagnosed in women between the 3rd and 5th decade, resulting in significant morbidity (7). The primary objective of SGS treatment focuses on establishing a sustained patent airway with preservation of a functional voice. Minimally invasive endoscopic procedures, including CO_2_/Nd:YAG laser incisions and resections, rigid and balloon dilations and (serial) intralesional steroid injections [(S)ILSI] are preferred first-line treatments, however, their benefits tend to be temporary and repeated treatments are often necessary (8, 9). Partial cricotracheal resection (PCTR) on the other hand offers the most durable results with a minimal long-term stenosis recurrence rate, but associated with a higher level of postoperative morbidity regarding complications and voice outcomes (10, 11). Moreover, only limited data exists on factors predicting restenosis after PCTR. Airway comorbidities [asthma, chronic obstructive pulmonary disease (COPD)], development of postoperative complications, craniocaudal extent of the subglottic/tracheal stenosis and the related length of resection and presence of glottic involvement have all been previously identified as potential risk factors for recurrent stenosis after PCTR (12, 13). This study aims at reporting and analysing complications and respiratory and vocal outcomes after PCTR.

## Patients and methods

### Compliance with ethical standards

The authors have no competing interests to declare that are relevant to the content of this article. No financial support was received from any organization for the submitted work. This monocentric retrospective cohort analysis was approved by and carried out in accordance with The Research Ethics Committee UZ/KU Leuven (study number MP023800) and with the ethical standards as laid down in the 1964 Declaration of Helsinki and its later amendments. The collection, processing and disclosure of personal data, such as participants' contact information, health information and medical information, are subject to compliance with the General Data Protection Regulation (GDPR).

### Patient selection

Patients who underwent airway surgery in the academic and tertiary referral University Hospitals of Leuven (Leuven, Belgium) between January 01, 2010 and December 31, 2022 were screened and patients were selected according to the following predefined criteria.

Inclusion criteria:

• Patients with diagnosis of SGS, with or without prior endoscopic procedures
• Treatment with PCTR with or without anterior thyrotomy
• Minimal age of 18 years

Exclusion criteria:

• Combined glottic-subglottic stenosis with glottic stenosis defined as bilateral decreased abduction of vocal folds causing significant restriction of the glottic lumen.
• PCTR performed for indications other than SGS (oncological indications)
• Previous history of external beam radiotherapy in the head and neck region
• Previous history of open surgery of the larynx or trachea, including procedures like tracheal sleeve resection or laryngotracheal reconstruction, but excluding prior tracheotomy.
• Pediatric cases

### Study endpoints

The primary objective of this retrospective cohort analysis was to evaluate respiratory and vocal outcomes after PCTR for treatment of SGS. Secondary endpoints were assessing postoperative complications and identifying potential risk factors for recurrent stenosis after PCTR.

### Data

Relevant data regarding patient (gender, age, comorbidities, smoking), stenosis (craniocaudal extension, distance between glottis and stenosis, stenosis grade, vocal fold mobility, histopathology), treatment (prior endoscopic procedures, concurrent need for anterior thyrotomy/laryngofissure during CTR), complications and outcomes [restenosis rate, vocal and respiratory outcomes (cfr infra)] were extracted from the electronical clinical reports of the included patients, pseudonymized and stored in a protected REDCAP database (REDCAP, Vanderbilt University, Nashville, USA).

Details on craniocaudal length of the stenosis, stenosis grade and distance between the glottic plane and the stenosis were extracted from direct laryngoscopy/suspension microlaryngoscopy or flexible laryngoscopy/bronchoscopy reports or images when available. When these data were unavailable, stenosis dimensions were independently measured on pre-operative CT-scans by 2 independent examiners (JM and LM). In case of discrepancy between measurements, consensus was reached by joint re-evaluation of all available data related to stenosis dimensions. Stenosis grade was quantified using the Cotton-Myer classification (grade 1: ≤50% obstruction of subglottic lumen; grade 2: 51%–70%; grade 3: 71%–99%; grade 4: 100%) (14).

Complications recorded include anastomotic dehiscence, postoperative fibrin deposits/granulation tissue formation at the anastomosis (with or without need for endoscopic intervention), requirement of postoperative tracheotomy, postoperative subcutaneous emphysema, wound infection, haemorrhage, vocal cord paralysis; unforeseen ICU admission, in-hospital mortality and development of a restenosis, which is defined as a recurrent and symptomatic airway obstruction after completion of the postoperative healing phase requiring surgical treatment (endoscopic procedures or redo open airway surgery).

Auditory-perceptual evaluation of voice quality was done by experienced speech and language pathologists using the GRBAS scoring system. Grade (G) represents the overall degree of hoarseness of the voice, roughness (R) represents an impression of the irregularity of vocal fold vibrations and breathiness (B) represents a psychoacoustic impression of the extent of air leakage through the glottis. Astheny (A) denotes weakness or lack of power in the voice and strain (S) represents a hyperfunctional state of the phonation heard (15). Subjective evaluation of the voice by assessing psychosocial impact of the voice quality as reported by patients was performed with the VHI-10 questionnaire, with higher scores indicating a higher level of vocal impairment (16). According to institutional guidelines, introduced in 2020, all SGS patients needing CTR undergo a complete vocal assessment based on the ELS protocol including the VHI-10 score preoperatively and 6 weeks after CTR (17). If patients experience suboptimal postoperative vocal outcomes, speech and language therapy is initiated and evaluation of vocal quality is repeated 3, 6 and 12 months postoperatively, depending on the duration of vocal complaints. If, at the time of data retrieval and manuscript preparation, included patients did not have a recent vocal assessment, they were contacted by phone for voice evaluation using the VHI-10.

Respiratory outcomes were evaluated by spirometry, including peak expiratory flow (PEF) and MIF50 at 6 weeks, 3 months, 6 months and 1 year after surgery and compared to pre-operative pulmonary function. In this paper, the most recent outcome measures are reported. PEF is the maximal flow during a short maximal expiratory effort after full inspiration; it reliably reflects the treatment outcomes in patients with SGS and can be utilized for longitudinal monitoring of individuals with SGS (18–20). MIF50 is defined as the flow rate during the middle of inspiration, measured at 50% of the forced vital capacity (FVC) (21).

### Treatment

Candidates for PCTR are submitted to a thorough pre-operative assessment, including CT scan of the head and neck region and the mediastinum, endoscopic examination (flexible laryngoscopy, flexible bronchoscopy or suspension microlaryngoscopy with assessment of the stenosis with rigid telescopes), blood testing (standard blood test with auto-immune disease screening including CRP, ANA, ANCA and serum IgG4) and functional evaluation of the voice and respiration (cfr. supra).

**Figure 2:**
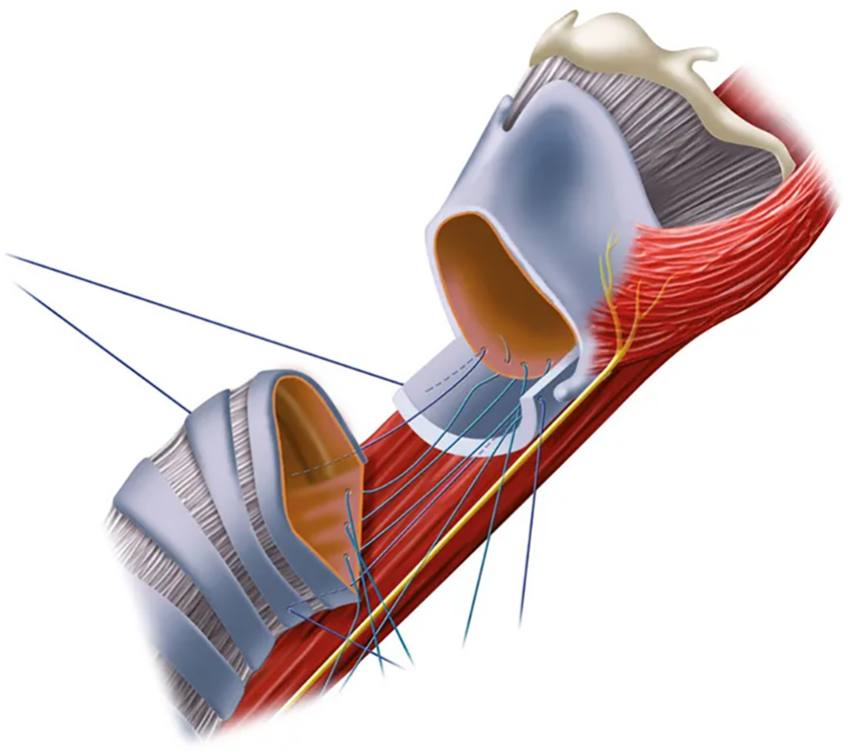
An anterolateral view of the cricotracheal anastomosis with placement of the sutures for the posterior cricotracheal anastomosis. Note the denudated posterior cricoid plate.

Surgical technique of PCTR has been described in detail elsewhere (22). Patients are ventilated with a laryngeal mask before and after resection of the stenosis and anastomosis, during which the laryngeal mask ventilation is switched to cross field endotracheal ventilation with intermittent apnoea. After a midline horizontal cervicotomy and division of the thyroid isthmus, progressive dissection down to the cricoid and first tracheal rings is performed. The trachea is bilaterally dissected with maximum preservation of its segmental blood supply. By careful dissection close to the tracheal cartilage, the recurrent laryngeal nerves are preserved without identifying them. Depending on the extent of the anticipated resection, progressive tracheal mobilization by mediastinal release, and less common laryngeal and/or hilar release is performed in order to achieve a tension free anastomosis. After detaching the cricothyroid muscle from its attachment on the anterior cricoid arch, horizontal incisions are made through the cricothyroid membrane just superior to the upper border of the cricoid and below the first tracheal ring, or lower depending on the extent of the stenosis into the trachea, hereby creating a dorsal mucosal flap of the membranous trachea, which will later be used to cover the cartilage of the cricoid backplate. The anterior cricoid arch and affected tracheal rings are resected, as well as all the mucosa and submucosa overlying the cricoid backplate. The posterior cartilaginous plate of the cricoid is preserved and a laryngotracheal anastomosis is performed while the patient is ventilated by cross-table ventilation with intermittent apnoea (Figure 2). When confronted with a relatively narrow immediate subglottic space cranially from the resection line, a subglottoplasty can be performed by removing submucosal tissue laterally and/or by suturing the lateral subglottic mucosa together with the conus elasticus to the inferior edge of the thyroid cartilage with a Vicryl or PDS 5/0, resulting in a widened subglottic airway. The anastomosis is started with an essential posterolateral “corner” suture on either side: a PDS 3-0 is passed through the posterolateral aspect of the second normal tracheal ring and through the posterolateral subglottic mucosa and the lateral aspect of the cricoid. Subsequently, the posterior anastomosis is performed using a running PDS 5-0 suture, achieving a perfect approximation between the dorsal mucosal flap of the trachea and the remaining posterior subglottic mucosa. Finally, the thyrotracheal anastomosis is completed by interrupted PDS 4-0 sutures. When confronted with a high extension of the SGS close to the vocal folds (<5–10 mm), a vertical anterior laryngofissure/thyrotomy exactly through the anterior commissure can be considered to facilitate the anastomosis. Flexible laryngoscopy through the laryngeal mask in the OR immediately after surgery and before awakening the patient is performed to evaluate the quality of the anastomosis and to assess the presence and extent of laryngeal edema and, related to this, the potential need for postoperative ICU stay and/or temporary tracheotomy. In routine cases, tracheotomy or stents (e.g., Montgomery T-tube) are avoided. Patients are immediately extubated in the OR and are advised to keep the neck in a flexed position for 1 week. A chin-to-chest suture is omitted in almost all cases. Flexible laryngoscopic evaluation is performed on postoperative days 1 (exclusion of vocal fold hematoma, excessive edema and vocal fold paralysis) and days 5 or 6 (exclusion of fibrin deposits on the anastomosis) and whenever the clinical situation necessitates evaluation of the larynx during hospital stay. These early laryngoscopic evaluations guide the postoperative management with regards to therapeutic bronchoscopy in case of formation of excessive fibrin deposits or corticosteroid administration in case of severe glottic edema. Postoperative assessment is typically organized 3 weeks after surgery (flexible laryngoscopy) and 6 weeks, 3 months, 6 months, 12 months and 24 months postoperatively (flexible laryngoscopy and functional evaluation). If definitive histopathological examination is suspect for underlying systemic disease [e.g., granulomatosis with polyangiitis or IgG4 related disease (IgG4 RD)], the patient is referred to internal medicine for further evaluation and systemic treatment.

### Statistical methodology

Statistical analysis was performed using SPSS (SPSS Inc, v. 29.0.1.0, IBM, Armonk NY, USA). Patient-, stenosis-, treatment and outcome-related characteristics were summarized with percentages for categorical variables and means with standard deviation for continuous variables. Pre- and post-operative PEF/MIF50 values and VHI-10/GRBAS scores were compared by paired samples *t*-test after normal distribution of data was confirmed by the Shapiro–Wilk test. The potential effect of several variables on restenosis probability was assessed; the odds ratio and associated *p*-values (with a 95% confidence interval) for the occurrence of restenosis and fibrin deposits/granulation tissue formation were determined through logistic regression. *P*-values <0.05 were considered statistically significant.

## Results

### Patient and stenosis characteristics

A total of 37 patients were included in this study. Mean age at the time of PCTR was 48.5 years (SD: 13.3, range: 25–80 years). Patients were predominantly female (*n* = 35, 94.6%) and all were non-smokers. Concerning comorbidities, a history of gastro-oesophageal reflux disease (GERD) as defined by proton pump inhibitor (PPI) use for GERD symptoms or GERD documented by esophagogastroscopy, was reported in the majority of patients (*n* = 29, 78.4%). Obstructive airway disease [asthma or chronic obstructive pulmonary disease (COPD)] and type II diabetes were present in 29.7% (*n* = 11) and 2.7% (*n* = 1) of patients respectively. The vast majority of patients (*n* = 33, 89.2%) had been pretreated with a median number of 4 endoscopic procedures (range: 1–9), including suspension microlaryngoscopy with radial CO2-laserincisions or -resections, flexible bronchoscopic CO_2_ Nd:YAG lasering, balloon dilatations and (serial) intralesional steroid injections (ILSI). Concerning etiology, most stenoses (*n* = 33, 89.2%) were considered to be idiopathic (iSGS). One patient (2.7%) was diagnosed with a post-intubation SGS and in 3 patients (8.1%), an underlying systemic disease (IgG4 RD) was deemed responsible for the stenosis. Stenoses were classified as Cotton-Myer grade III in 20 patients (54.1%), grade II in 13 patients (35.1%) and grade I in 4 patients (10.8%). The mean craniocaudal length of the stenosis was 17.5 mm (SD: 5.5, range: 5.5–37.0 mm) In 14 patients (38%), the stenosis extended cranially to less than 10 mm from the glottic level. Mean distance between the vocal folds and the cranial extent of the stenosis was 8.7 mm (SD: 5 mm). On pre-operative indirect laryngoscopic examination, vocal fold mobility was judged normal in 43 patients (91.9%); reduced vocal fold mobility was observed in 3 cases (8.1%). One patient (2.7%) had a permanent tracheostomy before surgery. Patients and stenosis characteristics are summarized in Table 1.

**Table 1:** Patient and stenosis characteristics.

Variable	Total (*n* = 37)
	*n* (%)
Sex	
Male	2 (5.4)
Female	35 (94.6)
Age at surgery (years) (SD)	48.5 ± 13.3
Smoker	
Yes	0 (0)
No	37 (100)
Comorbidities	
GERD	29 (78.4)
Diabetes mellitus	1 (2.7)
Astma/COPD	11 (29.7)
Etiology	
Idiopathic	33 (89.2)
Postintubation	1 (2.7)
Systemic disease	3 (8.1)
Pretreatment	
Endoscopic procedures	33 (89.2)
Number of endoscopic procedures (SD)	3.8 ± 2.1
Distance stenosis—vocal cords (mm)(SD)	8.7 ± 5
≥10 mm	23 (62)
<10 mm	14 (38)
Length stenosis (mm) (SD)	17.5 ± 5.5
Grade of stenosis	
I (0–50%)	4 (10.8)
II (51–70%)	13 (35.1)
III (71–99%)	20 (54.1)
IV (100%)	0 (0)
Vocal cord mobility pre-operative	
Normal	34 (91.9)
Abnormal/reduced	3 (8.1)

GERD, gastroesophageal reflux disease; COPD, chronic obstructive pulmonary disease; SD, standard deviation.

### Treatment characteristics and complications

Twenty-five (67.6%) patients underwent a standard PCTR, 12 (32.4%) patients with a high cranial extent of the stenosis had a supplemental anterior laryngofissure/thyrotomy in order to facilitate completion of the laryngotracheal anastomosis. Postoperative intensive care stay proved necessary in 3 (8.1%) patients, with a mean stay of 3.7 days (SD: 3.8, range: 1–8 days). Reasons for ICU stay were postoperative progressive stridor and respiratory distress due to laryngeal edema (*n* = 2), necessitating a more intensive monitoring and tracheostomy in 1 of them, and postoperative anastomotic dehiscence (*n* = 1) with need for surgical reintervention and tracheostomy (cfr infra). No significant relation between the need for additional anterior thyrotomy and postoperative ICU stay could be observed (fisher exact test, *p* = 0.2407). Postoperative flexible bronchoscopy was not performed on a routine basis, but was necessary in 8 (21.6%) patients who developed excessive fibrin deposits on the laryngotracheal anastomosis shortly after surgery, causing dypnoea. Average duration between surgery and therapeutic bronchoscopy, during which the fibrin deposits could be easily removed with cold instruments, was 4 days (range: 2–7 days). An additional 7 (18.9%) patients underwent a therapeutic bronchoscopy in a later postoperative phase (mean postoperative day 46, SD: 32, range: 16–119 days) for removal of abundant granulation tissue at the anastomotic site, which was typically observed on flexible laryngoscopy during routine follow-up. Postoperative subcutaneous emphysema was observed in 4 (11.4%) patients: in 3 patients the emphysema was mild with spontaneous resolution in the early postoperative phase and probably due to an anastomosis which was not completely air-tight; in 1 patient the sudden-onset severe emphysema was caused by an anastomotic breakdown, (cfr infra). Postoperative wound infection occurred in 3 (8.1%) patients and was treated by surgical exploration and drainage (*n* = 1) and antibiotic therapy (*n* = 2). No relation between postoperative wound infection and the presence of postoperative emphysema was observed (Fisher exact test, *p* = 0.313). Anastomotic breakdown and postoperative haemorrhage were both seen in 1 (2.7%) patient and both treated with surgical reintervention. The anastomotic breakdown was localized anteriorly due to necrosis of the anterior part of the tracheal stump with the posterior and lateral aspects of the anastomosis still viable and intact. The anterior tracheal necrosis was removed with preservation of the cricotracheal corner sutures and a partial anterior re-anastomosis with creation of a small tracheostoma at the level of the anastomosis was made. In one (2.7%) patient, a permanent unilateral vocal fold paralysis was observed. Two (5.4%) patients required a (temporary) tracheostomy in the early postoperative phase: 1 patient developed anastomotic breakdown necessitating reintervention with creation of a tracheostomy which was kept *in situ* for 53 days; the other patient required a tracheostomy for 41 days due to persistent stridor caused by laryngeal oedema and a hematoma between the cricoid plate and the posterior tracheal wall.

No treatment-related deaths were observed in our patient cohort. On definitive pathological examination, surgical specimens showed signs of atypical inflammation and fibrosis in 28 (75.7%) patients. Although serum IgG4 levels were elevated (>1.400 g/L) in only 2 patients, IgG4-positive plasma cells were detected in the surgical specimen in 9 (24.3%) patients, suggesting underlying IgG4 related disease. Treatment characteristics and complications are summarized in Table 2.

**Table 2:** Treatment characteristics and complications.

Variable	Total (*n* = 37)
	*n* (%)
Length of follow-up (days) (SD)	368.2 ± 358.9
Laryngofissure	
Yes	12 (32.4)
No	25 (67.6)
Restenosis	
Yes	2 (5.4)
No	35 (94.6)
Time after CTR until restenosis (days) (SD)	52 ± 19
Length restenosis (mm) (SD)	14 ± 6
Complications	
Anastomotic dehiscence	1 (2.7)
Tracheostomy	2 (5.4)
Duration tracheostomy (days) (SD)	47 ± 6
Sucutaneous emphysema	4 (11.4)
Post-operative haemorrhage	1 (2.7)
Infection	3 (8.1)
Conservative management	2 (5.4)
Surgical management	1 (2.7)
Vocal cord paralysis	1 (2.7)
Perioperative mortality	0 (0)
Endoscopic re-intervention	15 (40.5)
Granulation tissue	8 (21.6)
.CTR-endoscopy interval (days) (SD)	46.4 ± 31.8
Fibrin deposits	7 (18.9)
CTR-endoscopy interval (days) (SD)	3.9 ± 2.2
Mean number of endoscopic procedures (SD)	1.7 ± 0.8
ICU stay	3 (8.1)
Duration (days) (SD)	3.7 (1–8)
Histopathology	
Chronic inflammation, fibrosis	28 (75.7)
IgG4	9 (24.3)

CTR, cricotracheal resection; ICU, intensive care unit; IgG4, immunoglobulin G4; SD, standard deviation.

### Outcomes

The mean and median length of postoperative follow-up was 368 and 296 days respectively (SD = 359, range 20–1,697 days). The success rate of PCTR, defined as the achievement of a stable and patent airway without development of a recurrent symptomatic stenosis (other than fibrin deposits/granulation tissue in the early postoperative phase) necessitating additional surgical interventions, was 94.6%. Restenosis occurred in 2 (5.4%) patients. One patient developed extensive cicatrisation at the level of the posterior portion of the anastomosis 33 days after PCTR, which was successfully treated by 1 additional endoscopic laser procedure, resulting in a stable and fully patent airway as observed during bronchoscopy 2 months post-operatively. The second patient was diagnosed on postoperative day 71 with a granulating restenosis at the anterior and lateral portions of the anastomosis, which developed after a complicated postoperative phase with anastomotic dehiscence, necessitating revision surgery and creation of a tracheostoma which was *in situ* for 53 days. The restenosis was treated with 2 endoscopic laser resections and balloon dilatations with an interval of 26 days, eventually resulting in a fully patent airway.

**Figure 3:**
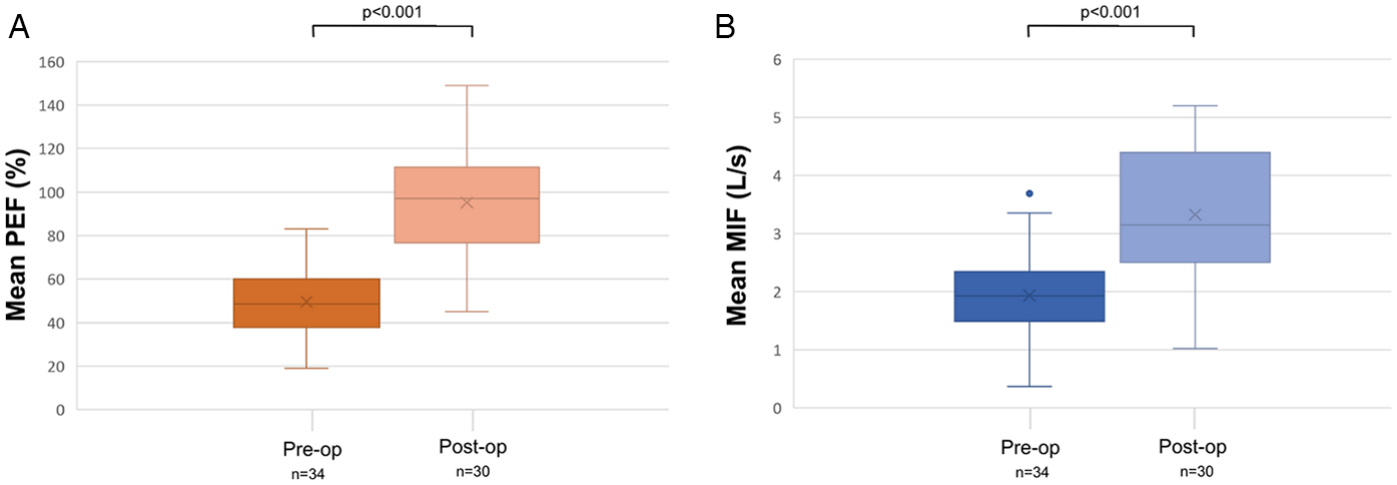
Pre- and postoperative evaluation of mean peak expiratory flow (in %) **(A)** and mean maximum inspiratory flow at 50% (in L/s) **(B)**. MIF: Maximum Inspiratory Flow; PEF %: Peak expiratory flow percentage.

Objective postoperative respiratory outcomes (PEF and MIF50) were available in 30 (81.1%) patients with a mean time interval between surgery and last PEF/MIF50 measurements of 230 days (SD: 232, range: 25–1,204). PEF increased significantly from 49.6 ± 15.4% preoperatively to 95.2 ± 23.5% postoperatively (*p* < 0.001), MIF50 improved from 1.9 ± 0.78 L/s to 3.3 ± 1.1 L/s (*p* < 0.001) (Table 3). Figure 3 depicts pre- and postoperative mean PEF (in %) (Figure 3A) and mean MIF50 (in L/s) (Figure 3B). Of the remaining 7 patients, lacking objective postoperative respiratory outcome measures, 5 reported complete resolution of dyspnoea, while the other 2 subjectively experienced mild exercise-induced dypnoea, however all of them without evidence of a recurrent subglottic stenosis on laryngoscopic examination and as such possibly attributable to physical deconditioning.

**Table 3:** Functional outcomes.

Variable	Mean ± SD
Spirometry	
PEF % pre-operative	49.6 ± 15.4 (*n* = 37)
PEF % post-operative	95.2 ± 23.5 (*n* = 30)
MIF50 pre-operative (L/s)	1.9 ± 0.8 (*n* = 34)
MIF50 post-operative (L/s)	3.3 ± 1.1 (*n* = 30)

MIF50, maximum inspiratory flow at 50%; PEF %, peak expiratory flow percentage; SD, standard deviation.

**Figure 4:**
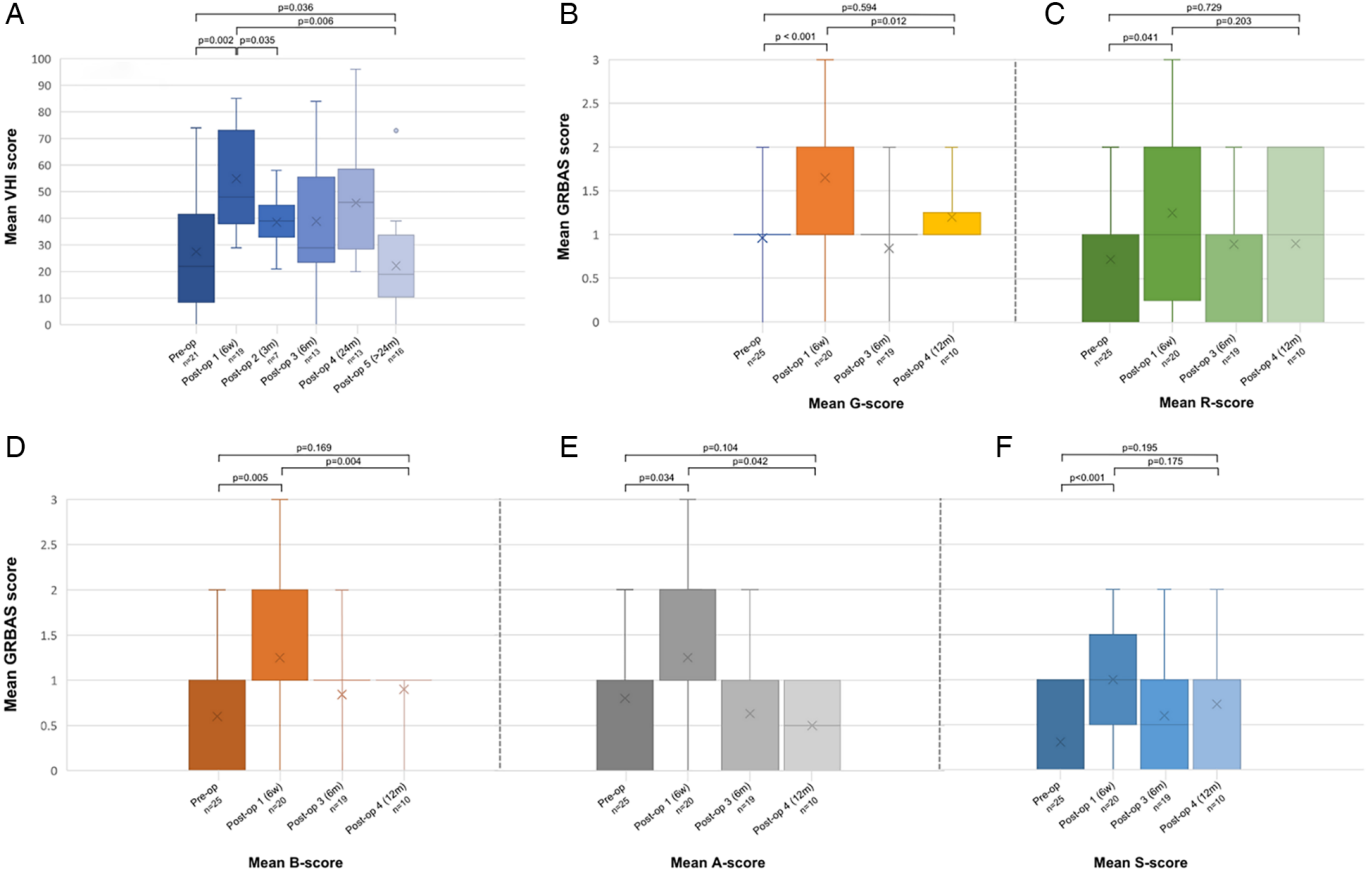
Pre-and postoperative evaluation of Voice Handicap Index **(A)**, grade of hoarseness **(B)**, roughness **(C)**, breathiness **(D)**, astheny **(E)** and strain **(F)**. A, astheny; B, breathiness; G, grade; R, roughness; S, strain; VHI, voice handicap index.

Concerning vocal outcomes, a significant increase in grade (G) of hoarseness (*p* < 0.001), roughness (R) (*p* = 0.041), breathiness (B) (*p* = 0.005), astheny (A) (*p* = 0.034) and strain (S) (*p* < 0.001) was observed 6 weeks after surgery when compared to pre-operative GRBAS scores. However, scores decreased to values comparable to the pre-operative scores 1 year after surgery (G: *p* = 0.594; R: *p* = 0.729; B: *p* = 0.169; A: *p* = 0.104; S: *p* = 0.195). In parallel, VHI-10 scores increased significantly from 27.5 ± 23.7 to 54.9 ± 18.7 (*p* = 0.002) at 6 weeks postoperatively and gradually decreased towards scores lower than pre-operative scores >24 months after surgery (22.2 ± 18.1, *p* = 0.036). Figure 4 depicts pre-and postoperative evaluation of Voice Handicap Index (Figure 4A), grade of hoarseness (Figure 4B), roughness (Figure 4C), breathiness (Figure 4D), astheny (Figure 4E) and strain (Figure 4F).

### Prognosticators for restenosis and fibrin deposits/granulation tissue formation

No potential prognostic effect of several variables (presence of GERD/asthma/COPD, CTR combined with laryngofissure/anterior thyrotomy, postoperative presence of fibrin/granulation on the anastomosis, previous endoscopic interventions, cranio-caudal stenosis length, etiology and stenosis grade) on restenosis probability could be identified on univariable analysis (Table 4). Additionally, no risk factors for fibrin deposits/granulation tissue formation on the laryngotracheal anastomosis could be identified among the variables analysed (presence of GERD/asthma/COPD, CTR combined with laryngofissure/anterior thyrotomy, previous endoscopic interventions, cranio-caudal stenosis length, etiology and stenosis grade) (Table 5).

**Table 4:** Risk factors for restenosis.

Variable	Restenosis (*n* = 2)	No restenosis (*n* = 35)	OR (95% CI)	*P* value
Reflux			0.3 (0.0–4.5)	0.348
Yes	1	28		
No	1	7		
Astma/COPD			Undefined	0.998
Yes	2	9		
No	0	26		
Laryngofissure			Undefined	0.998
Yes	2	10		
No	0	25		
Fibrin/granulation			Undefined	0.998
Yes	2	13		
No	0	22		
Cranio-caudal length			1.1 (0.9–1.4)	0.300
Etiology				
Idiopathic			Undefined	0.999
Yes	2	31		
No	0	4		
Postintubation			0.0	1.000
Yes	0	1		
No	2	34		
Systemic disease			0.0	0.999
Yes	0	3		
No	2	32		
Cotton-myer pre-operative				
Grade 1			10.7 (0.5–217.2)	0.124
Yes	1	3		
No	1	32		
Grade 2			0.0	0.999
Yes	0	13		
No	2	22		
Grade 3			0.8 (0.0–14.6)	0.906
Yes	1	19		
No	1	16		
Previous endoscopic intervention	Undefined	0.999		
Yes	2	31		
No	0	4		

COPD, chronic obstructive pulmonary disease; OR, odd's ratio.

**Table 5:** Risk factors for formation of fibrin deposits/granulation tissue.

Variable	Fibrin/granulations (*n* = 15)	No fibrin/no granulations (*n* = 22)	OR (95% CI)	*P* value
Reflux			0.6 (0.1–2.9)	0.540
Yes	11	18		
No	4	4		
Astma COPD			3.9 (0.9–17.4)	0.070
Yes	7	4		
No	8	18		
Laryngofissure			2.9 (0.7–12.3)	0.133
Yes	7	5		
No	8	17		
Cranio-caudal length			1.0	0.508
Etiology				
Idiopathic			0.2 (0.0–2.0)	0.171
Yes	12	21		
No	3	1		
Postintubation			Undefined	1.000
Yes	1	0		
No	14	22		
Systemic disease			3.2 (0.6–39.3)	0.358
Yes	2	1		
No	13	21		
Cotton-myer pre-operative				
Grade 1			0.5 (0.0–4.8)	0.511
Yes	1	3		
No	14	19		
Grade 2			0.5 (0.1–2.2)	0.376
Yes	4	9		
No	11	13		
Grade 3			2.4 (0.6–9.4)	0.208
Yes	10	10		
No	5	12		
Previous endoscopic intervention	2.2 (0.2–23.5)	0.511		
Yes	14	19		
No	1	3		

COPD, chronic obstructive pulmonary disease; OR, odd's ratio.

## Discussion

In this paper, we report favourable outcomes of PCTR in a patient population with benign SGS and which consisted mainly of women with iSGS. Our success rate of PCTR, defined as the achievement of a stable and patent airway without development of a recurrent symptomatic stenosis necessitating additional surgical interventions, was 94.6%. Restenosis occurred in 2 (5.4%) patients and was definitively resolved with additional endoscopic procedures. After a mean follow-up of 368 days, all patients showed a permanent restoration of the airway, illustrated by a significant improvement in PEF from 49.6 ± 15.4% preoperatively to 95.2 ± 23.5% postoperatively (*p* < 0.001). This demonstrates that even in patients encountering early failure of the surgical procedure, success can still be achieved without the need for open redo surgery. Additionally, safety of PCTR was illustrated by 0% peri-and postoperative mortality and a low incidence of significant postoperative complications, with anastomotic breakdown occurring in 1 patient (2.7%). Postoperative wound infection, managed either conservatively or with surgical intervention, occurred in 8.1% of patients, while postoperative subcutaneous emphysema developed in 11.4% of patients. However, no relation between wound infection and the presence of postoperative emphysema, due to (limited) air leakage at the anastomosis, was observed. Our results are consistent with published data: major series demonstrate long-term positive outcomes in over 90% of patients, with perioperative mortality rates below 1%–2%, restenosis rates between 0 and 11%, anastomotic dehiscence rates between 0% and 5% and surgical reintervention rates ranging from 0 to 3% (23–31). With 40.5%, the incidence of fibrin deposits/granulation tissue formation at the level of the laryngotracheal anastomosis requiring endoscopic removal is higher in our patient series than in the series of Patrick et al, who reported a 16% incidence of granulation tissue formation in patients after CTR (11). However, this could possibly be explained by the systematic and liberal implementation of postoperative flexible laryngoscopic evaluations of the laryngotracheal anastomosis and our low threshold for endoscopic removal of fibrin and granulations. The aforementioned postoperative outcomes of PCTR, both in our series and in the literature, underline its value as a definitive treatment option for (i)SGS patients, yielding significantly better long-term results compared to endoscopic procedures (10). In a systematic review, Lavrysen et al. reported a rate of restenosis after endoscopic interventions of 68% on average (3).

Moreover, we evaluated potential correlations between existing comorbidities, stenosis characteristics (including stenosis etiology) and prior endoscopic interventions on one hand and development of restenosis and fibrin/granulation tissue formation on the other hand. Although airway comorbidities (asthma, COPD, unilateral vocal cord paralysis), postoperative complications, resection length and start of the stenosis at the vocal cords were identified as risk factors for retreatment in prior series, the prognostic effect of these variables on restenosis rate could not be confirmed in our series (12, 13, 32, 33). This is potentially due to our limited population size and the low number of restenosis events. Moreover, we did not demonstrate additional significant risk factors, nor did we identify any risk factors associated with the development of fibrin deposits and/or granulation tissue formation.

A drawback of PCTR is its negative impact on postoperative voice outcomes, as reported in previous studies. In an in-depth analysis of pre-and postoperative functional outcome parameters of 45 patients receiving laryngotracheal surgery, Evermann et al. reported a decrease in fundamental vocal pitch (203 Hz vs. 150 Hz, *p* < 0.001) and dynamic voice range (23.5 ± 5.8 semitones vs. 17.8 ± 6.7 semitones, *p* < 0.001). Moreover, the assessment of the Roughness– Breathiness–Hoarseness score showed significant increases in roughness (*p* < 0.001), breathiness (*p* = 0.011) and hoarseness (*p* < 0.001) 3 months after surgery; on the nine-item VHI, an increasing impairment from 6 (0–22) to 14 (0–33) was observed (*p* < 0.001) (34). In our series, similar postoperative functional evolutions could be observed: a significant increase in grade (G) of hoarseness (*p* < 0.001), roughness (R) (*p* = 0.041), breathiness (B) (*p* = 0.005), astheny (A) (*p* = 0.034) and strain (S) (*p* < 0.001) was observed 6 weeks after surgery when compared to pre-operative GRBAS scores. However, GRBAS scores decreased to values comparable to the pre-operative scores 1 year after surgery. In parallel, VHI-10 scores were significantly increased 6 weeks postoperatively but gradually decreased towards scores even lower than pre-operative scores 24 months after surgery. Thanks to our long term functional follow-up, our data illustrate how patient-reported vocal outcomes improve after an initial postoperative deterioration, eventually leading to a voice which is considered by the patients being as good or even better compared to the preoperative voice. This was also observed by Compton et al. in a group of 33 patients undergoing CTR: VHI scores increased significantly from baseline preoperative scores (27.2 ± 22.7) to a mean value of 44.3 ± 25.6 (*P* < .001) 1-month postoperatively but decreased below preoperative scores after 2 years, although non-significantly (18.8 ± 11.9, *P* = .795) (35). A possible explanation for the initial increase in VHI are on one hand the detachment of the cricothyroid muscles from the anterior cricoid arc, restricting pitch elevation, and on the other hand postoperative oedema or scarification at the level of the anterior commissure when an anterior thyrotomy was performed. After time, oedema subsides and breathing improves with a decrease in dyspnoea, causing improvement of subjective and objective vocal parameters (34). Additionally, in some SGS patients with cicatricial strands to the vocal folds, PCTR could succeed in restoring a normal mobility of the vocal folds, leading to better postoperative vocal outcomes compared to the preoperative voice (34).

This study is subject to several limitations. First, due the limited follow-up interval, potential late-onset restenosis will not be identified. Patrick et al. stated that 5- and 10-year recurrence rates for CTR are too short to assess the efficacy of the procedure, as 2 of the 8 recurrences in their series occurred 12 and 14 years after CTR (11). Second, the retrospective design of this study imposes an inherent inclusion bias. This was countered by including all consecutively operated CTR patients. Additionally, missing pre-and postoperative data on respiratory and vocal outcomes, together with the relatively small sample size, decrease the power of statistical analysis and as such might limit the strength of our conclusions. To overcome these limitations, multicentric prospective studies with well-defined standardized outcome parameters are required and initiated, e.g., the multicenter European Society of Thoracic Surgeons (ESTS)-AIR database (36).

## Conclusion

PCTR is an efficient and safe treatment for (i) SGS, with low complication and restenosis rates. In our series, all patients showed a permanent restoration of the airway. Despite an initial postoperative vocal deterioration, vocal outcomes improve during the postoperative course. Multicentric prospective studies are needed to evaluate long-term outcomes of CTR in different populations and to identify prognostic factors for restenosis and complications.

## Data Availability

The raw data supporting the conclusions of this article will be made available by the authors, without undue reservation.
